# Detection of Gel-Forming Polymers via Calcium Crosslinking, Applied to the Screening of Extracellular Polymeric Substances Extracted from Biological Aggregates

**DOI:** 10.3390/gels9020157

**Published:** 2023-02-16

**Authors:** Abdo Bou-Sarkis, Etienne Paul, Elisabeth Girbal-Neuhauser, Nicolas Derlon, Yolaine Bessiere

**Affiliations:** 1 LBAE, Laboratoire de Biotechnologies Agroalimentaire et Environnementale (UPS, URU 4565), Université de Toulouse, Institut Universitaire de Technologie, 24 rue d’Embaquès, 32000 Auch, France; 2 TBI, Université de Toulouse, CNRS, INRAE, INSA, 135 avenue de Rangueil, CEDEX 04, 31077 Toulouse, France; 3 Department of Process Engineering, EAWAG, Swiss Federal Institute of Aquatic Science and Technology, CH-8600 Dübendorf, Switzerland

**Keywords:** hydrogels, gelling capacity, calcium crosslinking, aerobic granules, alginate-like exopolymers, extracellular polymeric substances

## Abstract

The valorization of biological aggregates through the extraction of hydrogel-forming polymers can enhance the economics and sustainability of various processes in which bacteria are involved in organic waste transformation, such as wastewater treatment. Achieving these goals requires the development of a method capable of detecting the presence of gel-forming polymers in complex mixtures containing biopolymers that are most often unknown and uncharacterized. A miniaturized screening method capable of detecting gelation via ionic crosslinking using only 1 to 3 mg of the tested samples (commercial molecules or extracellular polymeric substances, EPSs) is proposed. The method consists of calculating a percentage of reactivity (%R) through UV-vis spectra and determining the percentage of gel volume (%Vg) formed after the addition of calcium. Both factors were combined to give a gelling factor (GF), and the test was applied to pure commercial molecules (BSA, DNA, alginate (ALV), and a mixture of them), allowing the classification of the following solutions according to their gel-forming capacity: GF_(ALV)_ > GF_(ALV+DNA)_ > GF_(BSA+ALV+DNA)_ > GF_(BSA+ALV)_ > GF_(DNA)_ > GF_(BSA+DNA)_ > GF_(BSA)_. As a relevant tool for screening hydrogel-forming solutions, the method was applied to the EPS extracted from aerobic granular sludge. The EPS (0.5% *w*/*v*) had a GF of 0.16 ± 0.03, equivalent to approximately half of the GF of ALV (0.38 ± 0.02 at 0.5% *w*/*v*). The developed test pushes the limits of the existing gel-detection techniques because it allows for quicker, less consuming, and more informative gelation detection through the use of simple methods that do not require sophisticated equipment.

## 1. Introduction

Aerobic granular sludges are self-assembled spherical biofilms composed of micro-organisms embedded in an extracellular polymeric substances (EPSs) matrix. These biofilms are used in wastewater treatment and present many benefits over activated sludges [1]. Extracted EPS have the potential to be used in a wide variety of applications [2,3,4,5] mainly due to their capacity to form stable hydrogels with divalent cations, like calcium [6,7,8]. A hydrogel is defined as a crosslinked polymeric network that has the capacity to absorb water while maintaining resistance to dissolution. In this sense, hydrogels are swollen by the solvent forming a one-phase system, whereas precipitates are dense, solid structures that make two-phase systems [9,10]. The extraction of gel-forming EPSs from aerobic granular sludge is conventionally carried out by alkaline treatment, followed by acidic precipitation, giving a yield varying from 1.5% to 29% of organic matter [11,12,13,14]. This underlines the necessity for a gel formation screening method that is able to detect the presence of gel-forming polymers at the beginning of the extraction protocol and to follow their loss during extraction (if any).

Some of the gel-forming polymers extracted from aerobic granules were named alginate-like exopolysaccharides (ALEs) due to their functional and chemical similarities with alginate [7]. In the presence of CaCl_2,_ the gelation mechanism relies on calcium ions playing the role of a crosslinker through bridging two carboxylic acid functions (negatively charged) from adjacent alginate polymers, specifically through their guluronic acids [15,16]. Different functional groups are implied in the (ionic) crosslinking reactions of biomolecules with gel-forming capacities, such as primary amines, alcohols, thiols, carbonyls, and carboxylic acids [16]. In fact, it was proven that the ALEs extracted from aerobic granules, whatever their origin, are made of a wide variety of molecules, including polysaccharides, proteins, lipids, and humic acids, among other molecules [13,14]. Further fractionation steps are required to obtain the fractions enriched with gel-forming EPSs to disclose the chemical groups that are responsible for the gelation mechanisms. Therefore, the development of a miniaturized method that is able to detect gel-forming capacity with low quantities of polymers either in liquid raw extracts and/or in the enriched soluble fractions obtained through diverse fractionation techniques (liquid chromatography, selective precipitation, etc.) is required.

In literature, the detection of the gelling capacity of EPSs is carried out through different methods. The first technique consists of extruding concentrated EPSs in a calcium solution and checking if a gel bead forms, therefore giving only a qualitative (gel formation or not) appreciation of the gel-forming capacity [17]. A study carried out by Seviour et al. (2009) used a rheometer equipped with a Couette cell to follow the storage and loss modulus of the sample (224 mg per test) [18], necessitating the sampling of large volumes of aerobic sludge, which could be problematic, especially for lab-scale reactors, as well as the availability of a rheometer being necessary. The most used method is based on the formation of cylindrical gels through dialysis for 24 h with divalent cations and then measuring the deformation of the formed hydrogels by dynamic mechanical analysis to obtain the corresponding Young’s modulus [6]. This generally requires 10% (*w*/*v*) EPS solutions and, therefore, 40 mg for each test. Efforts have been made by Pagliaccia et al. (2022) to limit the consumption of EPSs through the optimization of the gels’ geometry, the concentration of the EPS solutions, and the calcium ions being used. It was found that at 0.1 M Ca^2+^, the minimal concentration of EPS required is 5% (*w*/*v*) to obtain stable hydrogels and, therefore, 13.5 mg per test. The dependency of the hydrogel stiffness on the crosslinker concentration has also been underlined [8].

This article aims to study the feasibility and performance of a miniaturized screening method using a simple technique (UV-visible spectroscopy) to measure the reactivity of the samples to calcium ions, with the goal of ensuring the availability of gelation screening methods to a wider audience of scientists and industrials through the use of simple techniques. The test aims to detect the presence of the molecules capable of interacting with calcium and forming gels and compare the gel formation capacity of different solutions. This objective will be attained through the development of the method using commercial standard molecules alone or in mixtures and then through its application through the different steps of the extraction protocol of EPS performed on granular sludge to check its efficiency in selecting gel-forming polymers. The test will help monitor the efficiency of gel-forming EPS extraction and will therefore allow the development of more targeted extraction protocols for a better recovery of gel-forming biopolymers [14]. On an industrial level, the test will allow for the examination of the richness of wastewater treatment plants with gelling polymers for valorization purposes.

## 2. Results and Discussions

### 2.1. Calcium Requirements for Gel Formation

The detection of the calcium requirements for gel formation was performed to ensure that the molecules capable of reacting with calcium have the necessary amount of calcium to form gels. This was carried out by comparing the UV-vis spectra of supernatants obtained for 1.5% *w*/*v* alginate with low viscosity (ALV) in various Ca^2+^ concentrations (0; 0.025; 0.05; 0.1; 0.2 M). ALV was chosen because its density of negative charges is very high in comparison with other natural biopolymers due to the alternation of mannuronic and guluronic monomers bearing a negative carboxylic charge.

**Figure 1:**
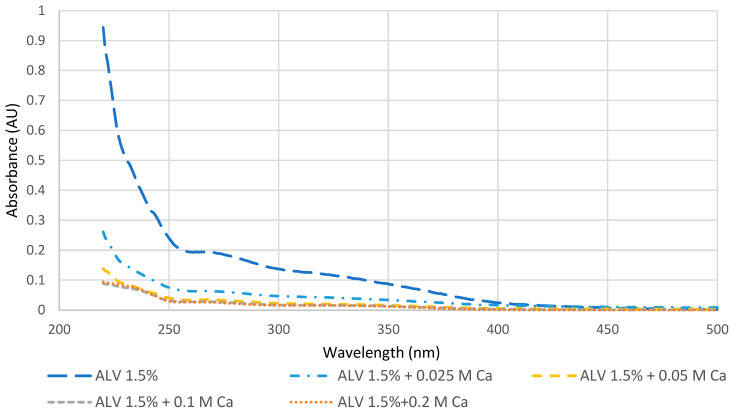
Effect of calcium concentration using UV-vis spectroscopy for ALV at 1.5% (*w*/*v*). For each calcium concentration, the spectrum shown is the average of three supernatant spectra obtained after gelation and includes the correction by the volume of supernatant/initial total volume (Vs/Vt).

Figure 1 presents the spectra with and without calcium. It is clearly noticed that ALV can react with small amounts of calcium since, even at 0.025 M, the spectrum decreased drastically because of gel formation. However, upon reaching 0.1 M in calcium, it can be seen that the spectrum stabilized and no further decrease in the signal was noticed in comparison with the addition of 0.2 M calcium. In agreement with this, other studies underlined that alginate solutions with concentrations varying between 1 and 5% were able to react even at very low concentrations of calcium (i.e., 0.001 M). However, the stiffness (Young’s modulus) of the formed alginate hydrogels at 2.5% *w*/*v* with 0.001 M calcium was approximately 2.3 times lower compared to the highest tested concentration of calcium (0.1 M) [8]. Therefore, the concentration of 0.1 M was chosen for the overall test.

### 2.2. Gelling Capacity of Standard Molecules Solutions

**Figure 2:**
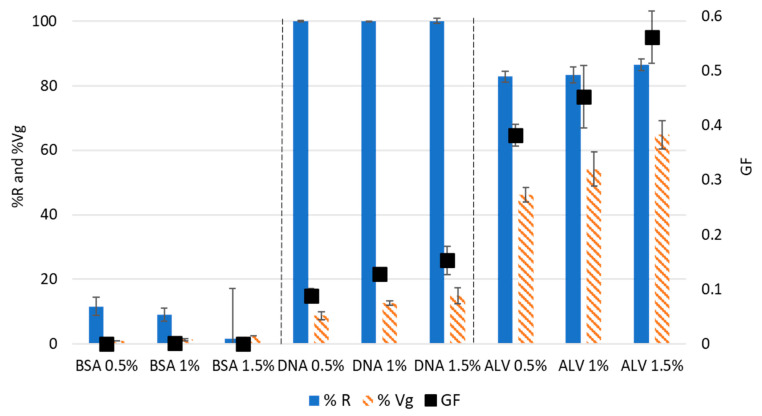
Effect of the polymer concentration (% *w*/*v*) of pure standard molecules on the %R, %Vg, and GF. The results are obtained through three gelation replicates and the standard deviations were represented as bars.

ALV, Bovine Serum albumin (BSA), and deoxyribonucleic acid (DNA) were used for the development of the test to represent the biochemical diversity within the EPS matrix. Whatever the tested polymer concentrations (0.5%, 1%, and 1.5% *w*/*v*), the BSA solutions exhibited a very low reactivity to calcium, with a %R of around 10% (Figure 2), and no significant differences were noted between the tested concentrations. After calcium addition, there was no or minimal decrease at the 280 nm wavelength specific to proteins, while a decrease in absorbance was specifically localized around the 220 nm wavelength (Supplementary Data Figure S1). This can be explained by changes in the secondary structure of BSA [19]. Another explanation could be the formation of Ca(OH)_2_ precipitates due to the interaction of calcium ions with the sodium hydroxide used to dissolve the samples. For the three tested BSA concentrations, the gel volume (Vg) was lower than 2.5% and did not increase significantly with an increase in BSA concentration (Figure 2), reinforcing the hypothesis that no gel formation occurred and that only a small pellet of Ca(OH)_2_ precipitate was formed. As expected, the GF of BSA (Figure 2) is very low, indicating that the solutions of BSA did not exhibit a measurable gelling capacity.

Concerning DNA, whatever the tested concentrations, all the molecules present in the solutions reacted with calcium, leading to %R values of around 100 % (Figure 2 and Figure S1). The %Vg parameter showed an increase from 8.3% to 15%, with an increase in the DNA concentration from 0.5% to 1.5% *w*/*v*. Hence, it can be noted that the GF of DNA increases with an increase in concentration to reach 0.15 ± 0.03 at 1.5% *w*/*v*. This capacity of DNA molecules to form gels can be explained by their backbone composed of negatively charged phosphates that allow for interactions with positively charged biomolecules and ions. Hence, nucleic acids can function as strong hydrophilic polyelectrolytes with the ability to absorb water. In the literature, DNA was described as an attractive material for hydrogel matrices due to its functional properties, such as biocompatibility, biodegradability, and permeability, among others [20]. In addition, the high affinity of DNA to calcium has been proven even in the presence of competing cations, such as monovalent cations (Na^+^ or K^+^) or divalent cations like Mg^2+^ [21].

The ALV solutions exhibited a high reactivity to calcium since the percentage of the initial area that disappeared was higher than 80% for all the tested concentrations (Figure 2 and Figure S1). In addition, a high percentage of gel volume was obtained, evolving from 46.1% to 68.1% with an increase in concentration from 0.5% to 1.5% *w*/*v* (Figure 2). The highest GF was noted for alginate at 1.5% (*w*/*v*) and was equal to 0.56 ± 0.04. Indeed, alginate is a well-known gelling molecule capable of forming gels with different ions and particularly with calcium [22]. Therefore, the capacity to form gels of the various solutions in terms of both molecular interaction with calcium and water swelling was as follows: ALV ˃ DNA ˃ BSA, whatever the tested concentrations.

### 2.3. Scanning Electron Microscopy (SEM) Characterization

**Figure 3:**
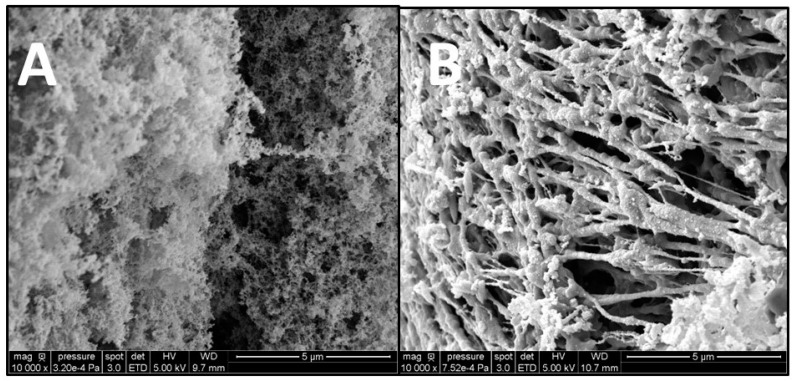
SEM observation of gels made of ALV (**A**), and DNA (**B**) at 1.5% (*w*/*v*) and 0.1 M Calcium.

The gels formed with Alginate and DNA were observed using SEM, and, in both cases, the 3D network responsible for gel formation was detected (Figure 3). However, different structures were observed; indeed, alginate hydrogels are more compact in comparison with DNA hydrogels that appear more filamentous with larger pores. The difference in porosity influences the properties of the hydrogels formed, such as their elastic properties. When compression is applied to a hydrogel, fluid is pushed through the pores, and the polymeric chains realign and then reach a point where the fluid’s ability to pass through the pores is reduced. Hydrogels with larger pores have a greater ability to recover once the mechanical load is reduced due to the greater ability of fluids to be redistributed within the polymeric network and, therefore, are more elastic [8].

### 2.4. Gelling Capacity of Mixtures of Standard Molecules

The developed method was tested on mixes of standard molecules, with the concentration of each molecule present in the final mixture set to 0.5% (*w*/*v*). This low concentration was chosen in order to evaluate the screening capacity for gelling molecules by respecting low material consumption and to check the capacity of the test to detect gelation, even in complex mixtures.

**Figure 4:**
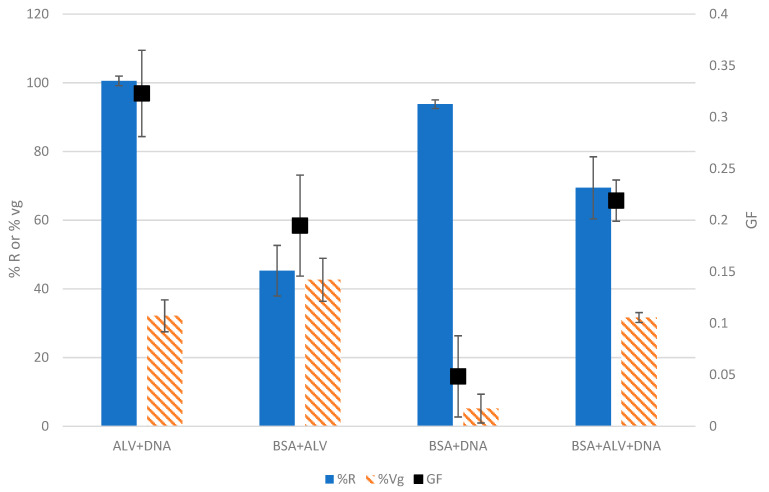
The %R, %Vg, and GF of the mixtures of the standard molecules, calibrated at 0.5% (*w*/*v*) for each molecule with 0.1 M calcium. The results are obtained through three gelation replicates and the standard deviations were represented as bars.

As shown in Figure 4 and Figure S2, the three solutions composed of two types of molecules exhibited GF values ranging from 0.05 ± 0.04 to 0.32 ± 0.04, the highest being obtained with the mixture of ALV+DNA and the lowest corresponding to the binary solution of BSA+DNA. The mixture containing the three standard molecules (BSA+ALV+DNA) had an intermediate GF of 0.22 ± 0.02 that decreased by one-third compared to the GF of the binary ALV+DNA solution (0.32 ± 0.04). This is consistent with the fact that BSA did not harm the capacity of ALV and DNA to form gels (%Vg remained the same between ALV+DNA and BSA+ALV+DNA). However, the addition of BSA to the mixture has reduced the ratio of the gelling molecules and, therefore, has caused a %R decrease in the tested conditions. In the same way, combining BSA with ALV reduced the GF by half, going from 0.38 ± 0.02 for ALV at 0.5% (Figure 2) to 0.19 ± 0.05 for the mixture of BSA+ALV. A similar reduction in the GF was also observed after the addition of BSA to a solution of pure DNA that decreased from 0.09 ± 0.01 to 0.05 ± 0.04.

Concerning the effects of DNA in the mixtures, it can be noticed that a decrease in %Vg is observed after the addition of DNA to a solution (from a %Vg of 46.1 ± 2.3% for ALV down to 32.1 ± 4.6% in the case of ALV+DNA). The same observation can be made upon comparing BSA+ALV with BSA+ALV+DNA. A hypothesis explaining this observation could be that DNA is a highly negatively charged molecule, which increases the negative repulsive forces between molecules in the solution, thus possibly hindering gelation. In addition, interactions between DNA and ALV could lead to the formation of a more compact hydrogel with a lower water retention capacity.

**Figure 5:**
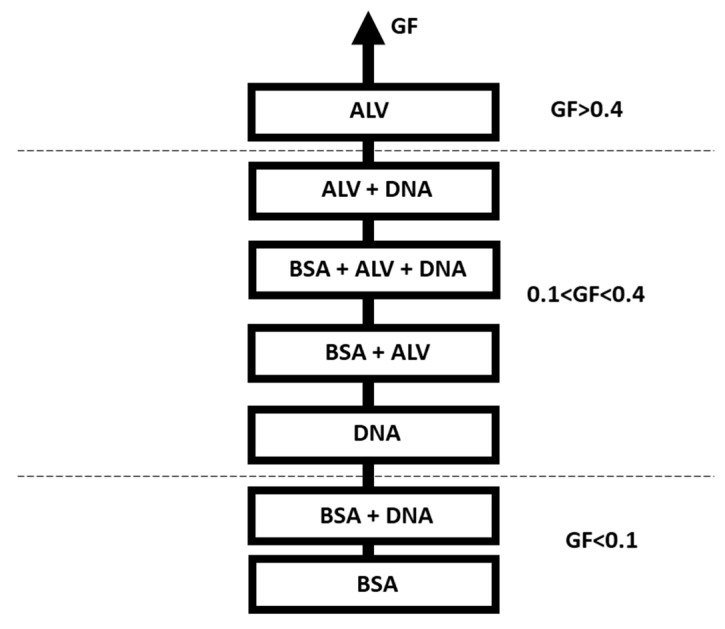
Classification of solutions according to their GF, %R, and %Vg.

In order to summarize the different cases when performing this test, a classification based on the GF, %R, and %Vg is proposed in Figure 5 and Figure S3. The previously tested solutions could be classified as samples with high gelling potential (GF > 0.4) resulting from a high %R and high %Vg or with low-gelling potential (GF < 0.1) or with an intermediate gelling potential (0.1 < GF < 0.4), divided into two categories depending on the %R (higher or lower than 50%) and the %Vg (higher or lower than 35%).

According to this classification, BSA and the binary of BSA+DNA are the representatives of the low GF group. As previously mentioned, BSA does not have a gel-forming capacity in the tested conditions. Although the %Vg of the binary BSA+DNA was similar to that of DNA, the binary BSA+DNA was classified as a low gel-forming solution because the ratio of gelling molecules present in the solution was reduced due to the presence of BSA (a non-gelling molecule). The high GF group consisted of the pure ALV solution, a gelling molecule with a high capacity for gel formation.

Particular attention should be paid to the solutions with a medium or intermediate GF, which could correspond to two categories. The first category is based on high %R (>50%) and low %Vg (<35%). In this case, the studied solution might be exhibiting precipitation behavior since it is reactive but does not have the capacity to retain water, which is a characteristic of hydrogels. Another possibility is that a gelling molecule that has a low water retention capacity is present in the solution. It can be noticed that the majority of solutions containing DNA are present in this category (as noticed previously, the presence of DNA reduced the %Vg hence decreasing the gel formation capacity).

The second profile that resulted from a low %R (<50%) but with a high %Vg (>35%) is due to a swelling behavior, therefore meaning the presence of a gelling molecule. In this case, the low %R might be due to a low extinction coefficient, therefore masking the reactivity of the gelling molecule. In this category, the BSA+ALV solutions are the only representatives. Indeed, as previously discussed, BSA did not hinder gelation, and this was previously shown in the literature where there was no influence of BSA on the post-gelling stiffness of alginate [8]. However, the presence of BSA reduced the ratio of gelling molecules in the solution, causing a decrease in %R. In addition, the extinction factors of BSA and ALV are very different, and this shows that even with quite different extinction factors, the test was still capable of detecting gelation. Attention must be paid when comparing solutions with the same GF since it can be possible to obtain a similar GF but with different %R and/or %Vg. This is clearly shown when comparing the BSA+ALV and BSA+ALV+DNA solutions that exhibited similar GF values (0.22 ± 0.02 and 0.19 ± 0.05, respectively) but different reactivities and gel volumes leading to different interpretations.

### 2.5. Application on Complex EPS Mixtures

**Figure 6:**
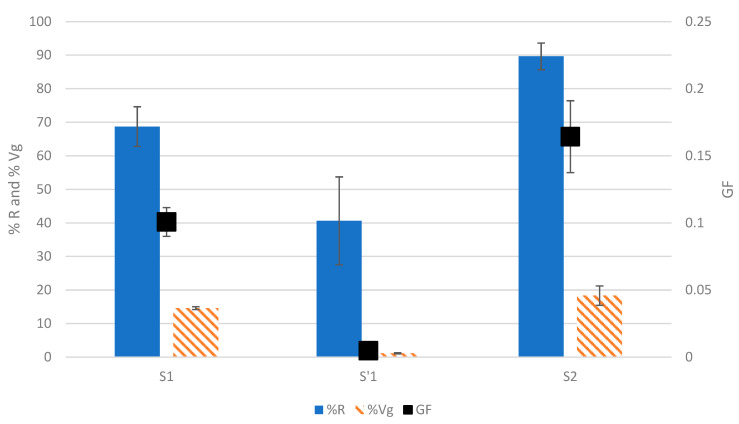
Comparison of the efficiency of the extraction protocol for the selection of gelling molecules using the developed method. The %R, %Vg, and GF are used to detect the capacity of solutions at 0.5% (*w*/*v*) and 0.1 M calcium to form gels. The results are obtained through three gelation replicates and the standard deviations were represented as bars.

The developed test was applied to the three EPS samples that were recovered during gel-forming EPS extraction from aerobic granular sludges [7,14]. The three tested fractions corresponding to the S1 (raw extract), S2 (solubilized acidic precipitate), and S’1 (supernatant of the acidic precipitation) were submitted to the test at a final concentration of 0.5% *w*/*v*, and the results can be seen in Figure 6 and Figure S4.

The highest GF was for S2 at 0.16 ± 0.03, followed by S1 at 0.10 ± 0.01, and finally, S’1 at 0.004 ± 0.002. The acidic precipitation is meant to select the gel-forming molecules [7,13,14], therefore explaining that the S2 solution had the highest GF. Since S1 is made by the totality of the extracted molecules (i.e., the molecules that have the capacity to form gels and those that do not), a lower GF was expected due to a lower ratio of gelling molecules in the solution. The gelling molecules are not supposed to be present in the supernatant of the acidic precipitation, S’1; hence, the lowest value of GF was obtained in this solution. Altogether, these results show that the protocol of extraction and acidic precipitation is indeed capable of extracting and concentrating the gelling molecules in one fraction (S2), where it can be noticed that the GF increased by a factor of 1.6 only through this step of acidic precipitation. As previously mentioned, biochemical groups often recurring in gel-forming EPSs are proteins, polysaccharides, and DNA [13,14,23]. However, they are not equally abundant in the mixture; for example, Bou-Sarkis et al. (2022) showed that there are equal amounts of proteins and polysaccharides in the EPS extracted using sodium carbonate, whereas it had approximately 40 times less DNA. Another study using anammox granules showed that EPSs are composed, to a lesser extent, of DNA in comparison with polysaccharides and proteins [24]. This means that the effects of DNA on the GF of S2 are most probably limited, but other contaminants were present, such as non-gelling proteins that could reduce the GF of S2, as was the case when mixing BSA with ALV.

The effect of the concentration on the GF values for S2 was quantified. As can be seen in Supplementary Data Figure S5A, in the tested range (0.5 to 1.5% *w*/*v*), the GF increased linearly (from 0.16 ± 0.03 to 0.23 ± 0.03).

A more filamentous and porous structure for S2 (Supplementary Data Figure S5B) was obtained in comparison with alginate gels (Figure 3). Pfaff et al. (2021) noticed that the gels formed by gel-forming EPSs are rather fibrous and filled with voids, and they attributed this observation to many factors, including the calcium concentration and the concentration of the gelling polymers; hence, these factors can be used to control the properties of the gels [25]. In 2022, Pagliaccia et al. attributed more significative linear elastic behavior to EPS hydrogels in comparison to alginate, and this could be due to a more porous structure.

### 2.6. Gelling Index: Comparing Solutions’Gelling Capacity to a Reference

Finally, a new parameter was proposed and named “gelling index”, with the goal of comparing the gelling capacity of a solution to an alginate solution at the same concentration (a well-known gelling molecule). This parameter will allow for the determination of the gelling capacity in reference to a well-studied gelling polymer and hence obtain a more tangible notion of the gelling capacity of the solution being studied in comparison with that of alginate.

**Figure 7:**
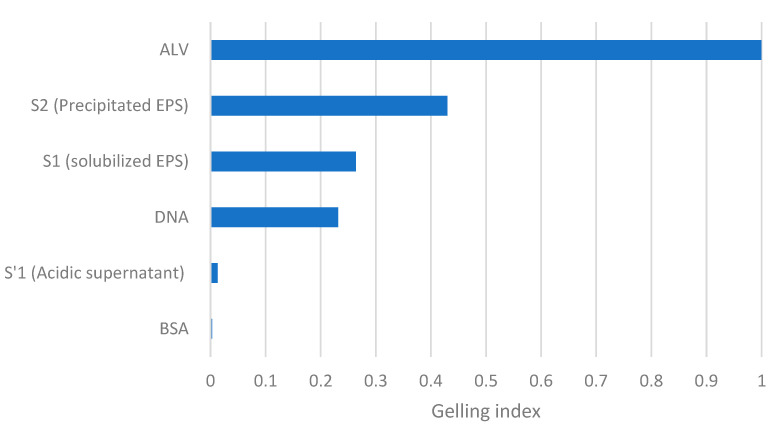
The GI of different solutions at 0.5% (*w*/*v*) with 0.1 M calcium, allowing for a comparison of their gelling capacities (between each other and to a reference molecule ALV).

The GI of both the standard molecules and the EPS samples described in this study was calculated for the concentration of 0.5% (*w*/*v*). As shown in Figure 7, the evaluation of the GI allows for the classification of solutions according to their capacity to form gels. It can be noticed rapidly that none of the solutions’ gelling index is close to 1 (alginate). Among the compared solutions, precipitated EPS in S2 has the highest gelling index: 0.43, followed by the S1 sample containing alkaline extracted EPS, with a GI of 0.26. This technique allows for a quick comparison between solutions’ capacities to form gels by using a reference (ALV). Indeed, when compared to the obtained results within the literature, it can be noticed that alginate has a better gel formation capacity than EPS; in particular, the value of alginate’s Young’s modulus is approximately 10 times higher than that of EPS at 5% *w*/*v* [8]. In addition, it was shown that alginate (at a concentration of 2.5% *w*/*v* compared to EPS at 10% *w*/*v*) had a Young’s modulus approximately 15 times higher than calcium [6]. Thus, Young’s modulus has a better sensitivity to detect the differences between hydrogels, but the proposed test remains interesting since it can provide information about the presence of nongelling molecules within a mixture and can be coupled with other methods to have a global view on the gelation capacity of a solution. The lower sensitivity compared to Young’s modulus is acceptable since the primary purpose of the developed test is to screen for the gelling capacity. In comparison to the literature [6,8,17,18], this screening method allows for reduced sampling from bioreactors due to the reduced amount of EPS needed per test, and offers a quick screening using simple equipment. These results show that the test is capable of detecting gel formation as a screening method to select interesting solutions for further characterization. The test showed that more purification of the extracted EPS is needed in order to obtain a solution of polymers with more interesting properties closer to that of alginate.

Therefore, the proposed test (Supplementary Data Figure S3) allows for a quick comparison of the gelling potential of the biological samples assayed at the same final concentration. The test can be used to monitor the increase or decrease in gelling capacity during the purification of biopolymers in order to quickly identify the relevant fractions. In addition, activating or inhibiting effects could be detected, thus offering the possibility of formulating new preparations or mixtures of biopolymers. In fact, the mixtures of molecules that give composite hydrogels were shown previously in the literature to have interesting properties. Hydrogels made by using xanthan gum and β-lactoglobulin have been described in which β-lactoglobulin plays the role of a crosslinking agent between the xanthan polymers when the pH conditions are convenient [26]. Hence, this test can be used to study composite hydrogels since it proved its capacity to detect gelation even in complex mixtures.

## 3. Conclusions

A method was developed to quickly screen the capacity of solutions to form gels in the presence of calcium. This method can be used to understand the role of contaminants in the hydrogel formation and by being coupled with microscopic observations (SEM), can provide knowledge on the structure of the hydrogel network formed. The method has the advantage of being less material and time-consuming in comparison to the conventional methods used in the literature and allows for the detection of gelation through the use of simple methods (UV-vis spectroscopy). Screening tests can be carried out using only 1 mg of the sample to be tested, leading to less sampling volumes and, therefore, less perturbation of lab-scale reactors.

The direct application of the method will be as a screening process for gelling capacity and to compare extracts’ capacities of gelation. The method was applied to the EPS extraction protocol as a proof of concept, and it was found that the protocol allowed for the selection of gel-forming molecules, but the obtained gelling solution still needs further purification to attain a GF closer to alginate and, therefore, a GI closer to 1. This test can be further used to follow the enrichment in gelling polymers during purification/fractionation processes. In conclusion, due to its ease of application, low consumption of materials, and speed, this test can be very helpful in the progression toward efficiently selecting gel-forming molecules from aerobic granules. In the future, trying to detect the participation of specific biochemical groups in gelation through the coupling of this test with fluorescent detection using specific probes can be tested. Moreover, this test can help in modulating and understanding the parameters affecting the gelation of different solutions, such as pH, divalent ions, the concentration of polymers, etc., and hence allow for the more efficient valorization of EPS.

## 4. Materials and Methods

### 4.1. Samples Preparation

Since the EPSs extracted from microbial biofilms or aggregates are made of a majority of polysaccharides and proteins [13,23], a representative polymer was selected for each group. Alginate with low viscosity (ALV) from brown algae (9005-38-3, Sigma-Aldrich, St. Louis, USA) was selected for the polysaccharide group because gel-forming molecules extracted from EPSs were found to exhibit similar properties to alginate, as previously explained [7]. BSA (9048-46-8, Sigma-Aldrich, St. Louis, USA) was chosen as a representative for the protein group since it is a well-studied protein that is known to be rich in ionic amino acids (like glutamic acid, which is negatively charged) [19] and does not have a gel-forming capacity unless it is heated beforehand [27,28], and was therefore chosen as a negative control for the gelation test. Since DNA is also commonly found in the alkaline extracts of granular sludges [14,23] it was also used. Indeed, DNA is a highly negatively charged polymer, which goes in favor of an interaction with calcium ions. Therefore, commercial DNA from salmon testes (438545-06-3, Sigma-Aldrich, St. Louis, MO, USA) was elicited as a representative molecule possibly involved in the gelation process of complex biological samples. The volatile solids (VSs) contents were determined for the powder of each commercial molecule, and they were dissolved in 0.1 M sodium hydroxide at final VSs concentrations of 3%, 2%, and 1% (*w*/*v*). Mixtures by pairs of commercial molecules (BSA+ALV, BSA+DNA, ALV+DNA) were prepared to obtain solutions at 2% (*w*/*v*) VSs concentration, with a ratio of 1:1 for each molecule, and a mixture of the three molecules (BSA+ALV+DNA) was also prepared so to obtain a solution of 3% (*w*/*v*) with a ratio of 1:1:1.

Finally, the test was applied to the three solutions recovered when performing the extraction of gel-forming EPS from aerobic granules, as explained in Bou-Sarkis et al. (2022) and summarized in supplementary data (Figure S6). Briefly, S1 contains all the polymers solubilized in 0.2 M sodium carbonate after the incubation of freeze-dried aerobic granules for 1 h at 80 °C; S’1 was the supernatant after the acidic precipitation of S1 raw extract, and S2 was the solution containing the gel-forming EPS corresponding to the acidic precipitated polymers. All the fractions were dialyzed against distilled water for 36 h and were freeze-dried. Samples were solubilized in sodium hydroxide 0.1 M at final VSs concentrations ranging from 1% to 3% (*w*/*v*) and were submitted to the test.

### 4.2. Test Methodology

A volume of 100 µL of the sample obtained (as described in Section 4.1) was diluted by a factor of two through the addition of either 100 µL of distilled water (sample without calcium) or 100 µL of 0.2 M calcium chloride (sample with calcium), leading to obtaining a final solution with 0.1 M CaCl_2_ at the desired concentration of the tested molecule (1.5%, 1%, and 0.5% *w*/*v*). The sample was homogenized by a vortex to ensure homogeneous calcium and polymer distribution. The incubation time was set to 1 h since it was shown in the literature that this time is sufficient for the formation of hydrogels [29]. All the samples were then centrifuged at 14,000× *g* for 15 min. The supernatants were recovered, and their volumes (Vs) were measured, allowing us to obtain the volume of the remaining pellet (Vg), i.e., the formed gel corresponding to the difference between the initial total volume of liquid (Vt) and the volume of the supernatant, which is the liquid remaining above the gel (Vs). A dilution of the supernatant should be carried out directly after centrifugation to avoid the formation of Ca(OH)_2_ precipitate. The lowest dilution, allowing for the obtainment of a value of absorbance between 0 and 1 (for the sample without calcium), was adopted for both samples (with and without calcium). A volume of 200 µL of each diluted supernatant was put in a 96-well UV transparent microplates (Microtiter), and a UV-visible spectrum was acquired between 220 and 500 nm using the nanoSpectrostar spectrophotometer (BMG-LABTECH). All the absorbance values (Absorbance Units AU) were multiplied by Vs over Vt to take into consideration the liquid volume change due to gelation after the addition of calcium. A sodium hydroxide spectrum was also measured in triplicate at the used dilution and was subtracted from the spectra of samples performed with and without calcium. All samples were analyzed in triplicates, and their averages were reported with the standard deviations.

### 4.3. Evaluation of Gel-Forming Capacity

To determine the percentage of reactivity to calcium, the trapezoidal method (with a step of 1 nm) was used to calculate the area below each spectrum, and the % of reactivity (%R) was obtained through Equation (1):

(1) $$\%\;R=\frac{\left(\mathrm{Vt}*\mathrm{AIS}-\mathrm{Vs}*\mathrm{ACaS}\right)*100}{\mathrm{Vt}*\mathrm{AIS}}$$

where AIS is the cumulated area of the initial spectrum without calcium [AU.nm], ACaS is the cumulated area of the spectrum with calcium [AU.nm], Vt is the initial total volume [µL], and Vs the liquid volume after calcium addition (supernatant after centrifugation) [µL].

The % of apparent gel volume (%Vg) was obtained by Equation (2):

(2) $$\%\;\mathrm{Vg}=\frac{\left(\mathrm{Vt}-\mathrm{Vs}\right)*100}{\mathrm{Vt}}$$

This parameter will allow for the distinction between a precipitate and a gel since precipitates do not usually tend to adsorb water and swell (contrary to hydrogels).

Using these two parameters, a gelling factor (GF) was proposed to quantify the capacity of a solution to form gels and compare them with each other (see Equation (3)):

(3) GF=%R∗%Vg

Finally, the gelling index (GI), defined in Equation (4), was used to compare the relative capacity of a solution to form hydrogels with a model gel-forming molecule (here ALV) in similar tested conditions:

(4) $$\mathrm{GI}=\frac{\mathrm{GF}\;\mathrm{of}\;\mathrm{the}\;\mathrm{sample}}{\mathrm{GF}\;\mathrm{of}\;\mathrm{ALV}}$$

### 4.4. Scanning Electron Microscopy (SEM)

After the gelation protocol (see Section 4.2), the obtained gel pellets were washed with distilled water and then dehydrated using ethanol. After critical-point drying, the samples were mounted on microscope stubs and sputtered with platinum (8 nm, 1 min, 60 mA). Samples were examined on an FEI Quanta 250 FEG scanning electron microscope at an accelerating voltage of 5 KV.

## Data Availability

All data generated or analyzed during this study are included in this published article, and the supplementary material available online with it.

## Supplementary Materials

The following supporting information can be downloaded at: https://www.mdpi.com/article/10.3390/gels9020157/s1, Figure S1: UV-vis spectra of BSA (A), ALV (B), and DNA (C) at 0.5% *w*/*v* with and without calcium at 0.1 M with blue line for polymer alone and orange line for polymer with calcium; Figure S2: Uv-vis spectra of mixtures at 1% *w*/*v* final concentration of ALV+DNA (A), BSA+DNA (B), BSA+ALV (C), BSA+ALV+DNA (D) with and without calcium at 0.1 M with blue line for polymer alone and orange line for polymer with calcium; Figure S3: Summary of the proposed test, with case studies of the GF; Figure S4: UV-vis spectra at 0.5% *w*/*v* of S1(A) S’1(B) S2(C) with and without calcium at 0.1 M; Figure S5: The effect of the concentration % (*w*/*v*) of the solutions of S2 (precipitated EPS) on the GF (A) and SEM observation of a 1.5% (*w*/*v*) with 0.1 M calcium S2 hydrogel (B).Figure S6: Summary of the extraction protocol of gel-forming EPS.

## Abbreviations

%R	percentage of reactivity
%Vg	percentage of gel volume
ACaS	Cumulated Area of the Spectrum with calcium
AIS	Cumulated Area of the Initial Spectrum without calcium
ALE	Alginate Like Exopolysaccharides
ALV	Alginate Low Viscosity
ALV+DNA	Alginate Low Viscosity with Deoxyribonucleic Acid
AU	Absorbance Units
BSA	Bovine Serum Albumin
BSA+ALV	Bovine Serum Albumin with Alginate Low Viscosity
BSA+DNA	Bovine Serum Albumin with Deoxyribonucleic Acid
BSA+ALV+DNA	Bovine Serum Albumin with Alginate Low Viscosity and Deoxyribonucleic Acid
DNA	Deoxyribonucleic Acid
EPS	Extracellular Polymeric Substances
GI	Gelling Index
GF	gelling factor
R	reactivity
S1	raw extract
S’1	supernatant after acidic precipitation
S2	solubilized acidic precipitate
SEM	Scanning Electron Microscopy
UV-vis	ultra violet-visible
Vg	gel volume
VS	Volatile Solids
Vt	initial total volume
Vs	liquid volume after calcium addition (supernatant after centrifugation)
