# Supplementary material for: Detection of Gel-Forming Polymers via Calcium Crosslinking, Applied to the Screening of Extracellular Polymeric Substances Extracted from Biological Aggregates
Source: Gels. 2023 Feb 16;9(2):157. doi: 10.3390/gels9020157 (PMC9957232; doi:10.3390/gels9020157)
Supplement: Supplementary file 1 [file gels-09-00157-s001.zip › gels-2212913-supplementary.pdf]

Supplementary data for:

Detection of Gel-Forming Polymers via Calcium Crosslinking, Applied to the Screening of Extracellular Polymeric Substances Extracted from Biological Aggregates

Abdo Bou-Sarkis 1,2,*, Etienne Paul 2, Elisabeth Girbal-Neuhauser 1,* , Nicolas Derlon 3 and Yolaine Bessiere 2

[1] LBAE, Laboratoire de Biotechnologies Agroalimentaire et Environnementale (UPS, URU 4565), Université de Toulouse, Institut Universitaire de Technologie, 24 rue d'Embaquès, 32000 Auch, France

[2] TBI, Université de Toulouse, CNRS, INRAE, INSA, 135 avenue de Rangueil, CEDEX 04, 31077 Toulouse, France

[3] Department of Process Engineering, EAWAG, Swiss Federal Institute of Aquatic Science and Technology, CH-8600 Dubendorf, Switzerland

* Correspondence: abdo.bou-sarkis@iut-tlse3.fr (A.B.-S.); elisabeth.neuhauser@iut-tlse3.fr (E.G.-N.)

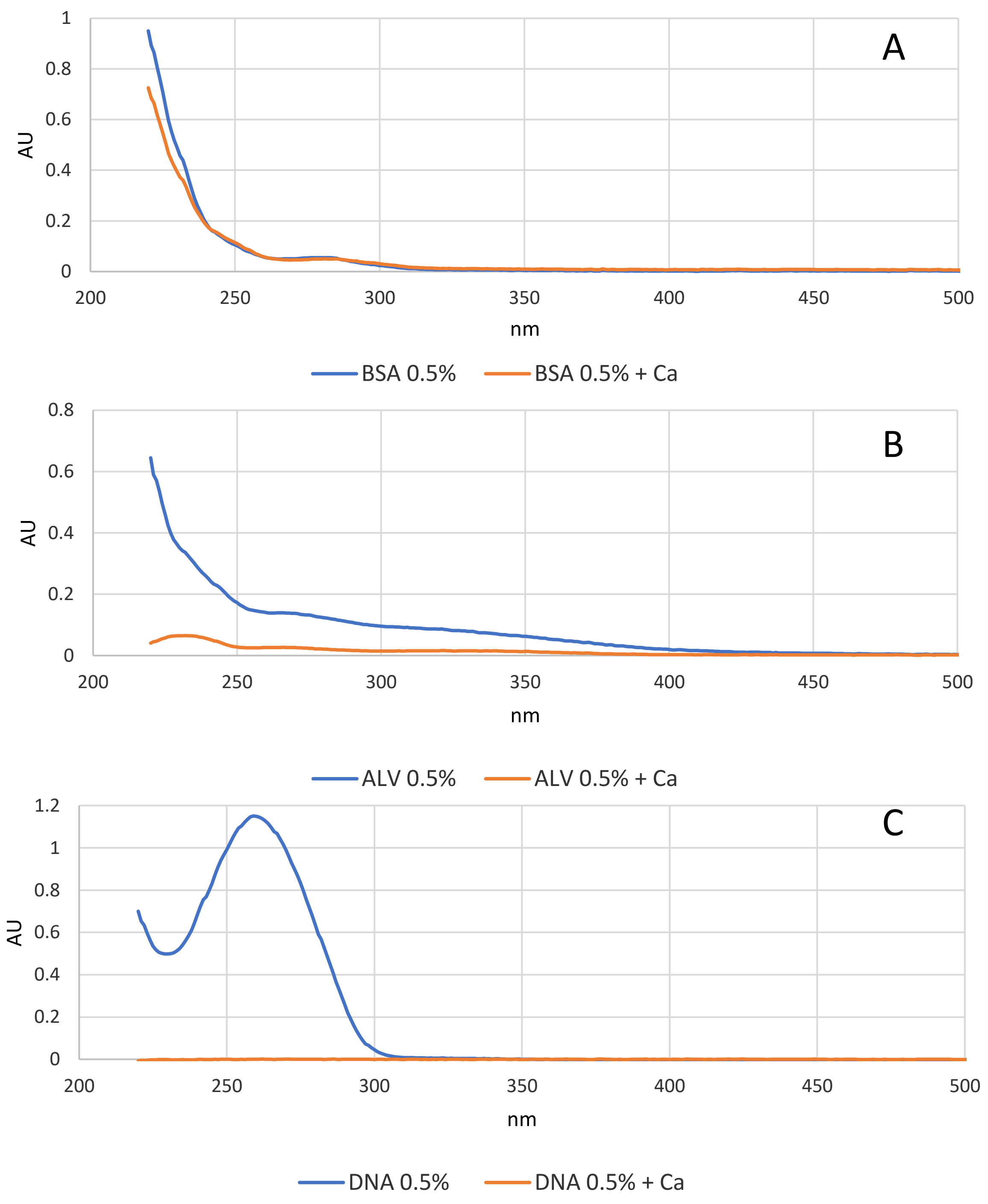

Figure S1: UV-vis spectra of BSA (A), ALV (B) and DNA (C) at 0.5% w/v with and without calcium at 0.1M with blue line for polymer alone and orange line for polymer with calcium.

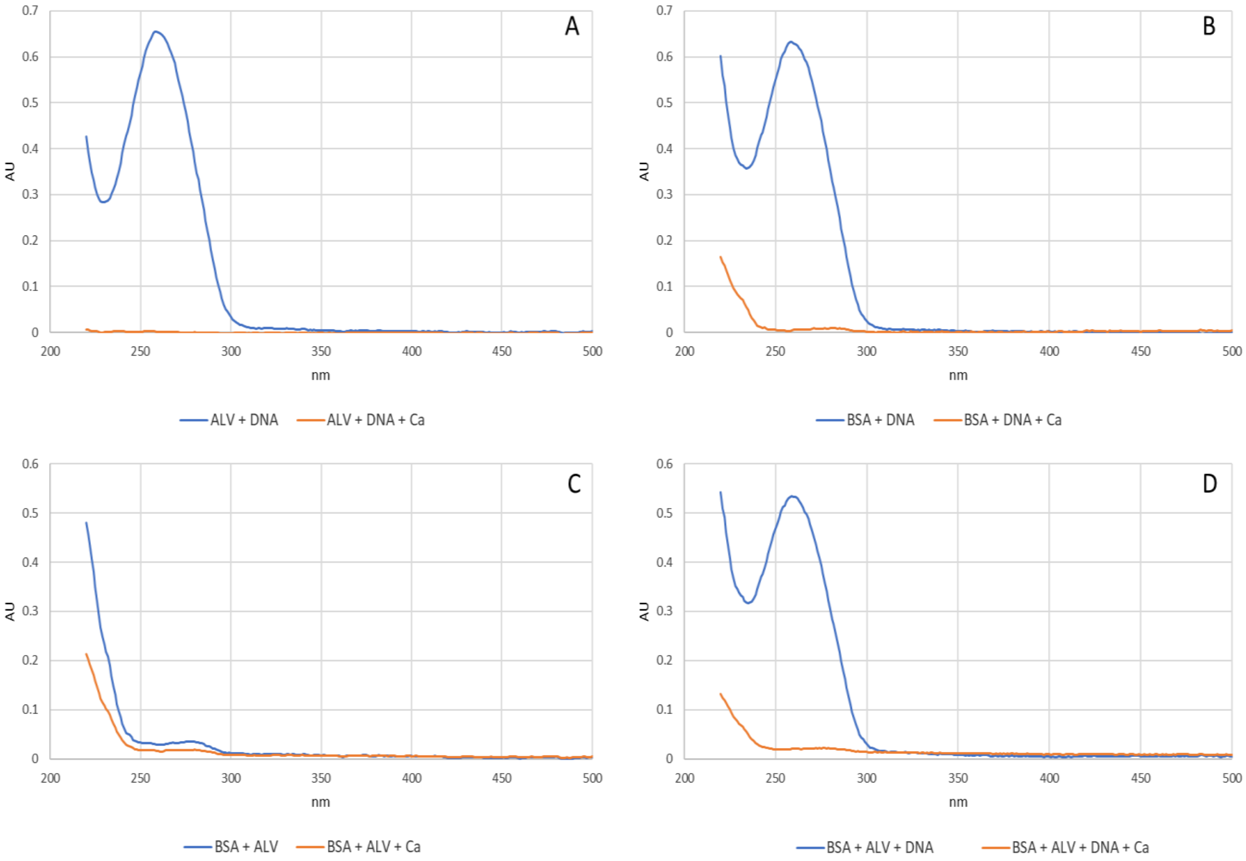

Figure S2: Uv-vis spectra of mixtures at 1% w/v final concentration of ALV+DNA (A), BSA+DNA (B), BSA+ALV (C), BSA+ALV+DNA (D) with and without calcium at 0.1M with blue line for polymer alone and orange line for polymer with calcium.

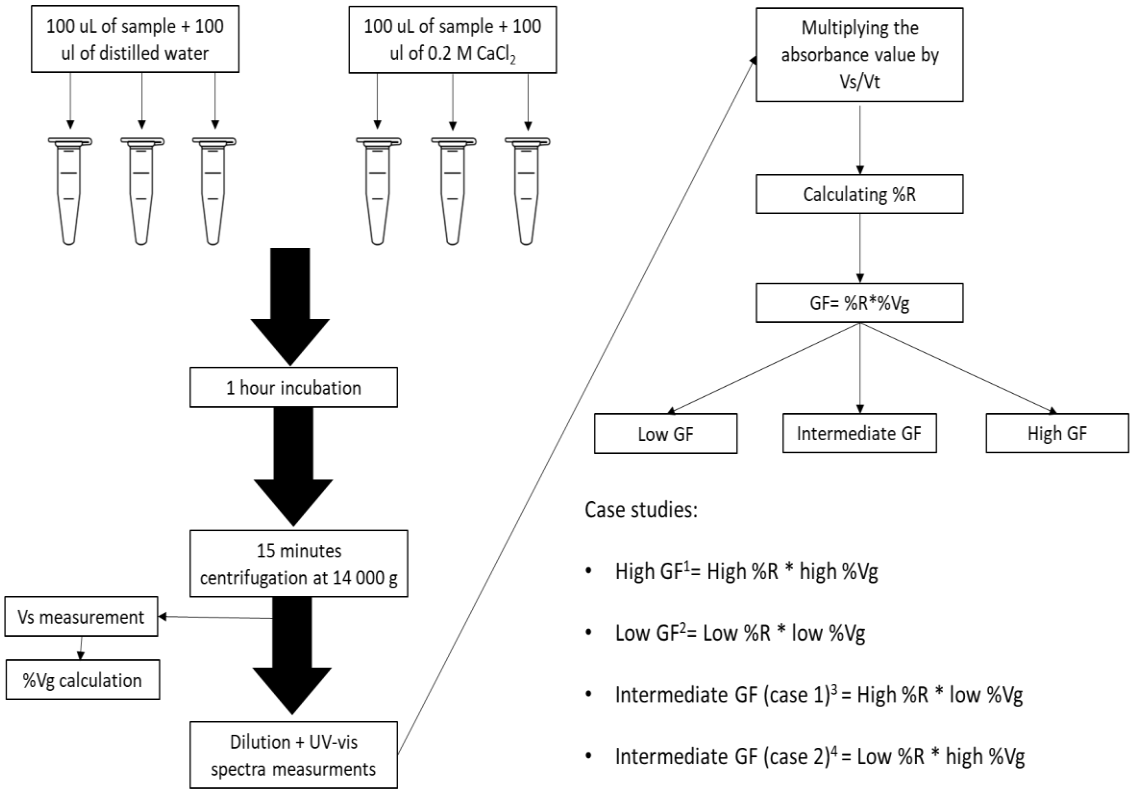

Figure S3: Summary of the proposed test, with case studies of the GF.

[1]: in the case of a high GF>0.4, resulting from a high %R and high %Vg, the solution being studied has a good gelation capacity such as ALV

[2]: in the case of a low GF<0.1, resulting from a low %R and low %Vg, the solution being studied has no gelation capacity such as BSA

[3]: in the case of an intermediate GF (0.1<GF<0.4), resulting from a high %R (>50%) and low %Vg (<35%), the solution being studied might be exhibiting a precipitation behavior or a gelling molecule that has low water retention capacity such as DNA, or a molecule with a high extinction coefficient and a small quantity in the mixture

[4]: in the case of an intermediate GF (0.1<GF<0.4), resulting from a low %R (<50%) and high %Vg (>35%), the high %Vg is due to a swelling behavior therefore meaning a gelling molecule present, the low %R in this case is due to a low extinction coefficient therefore masking the reactivity of the gelling molecule.

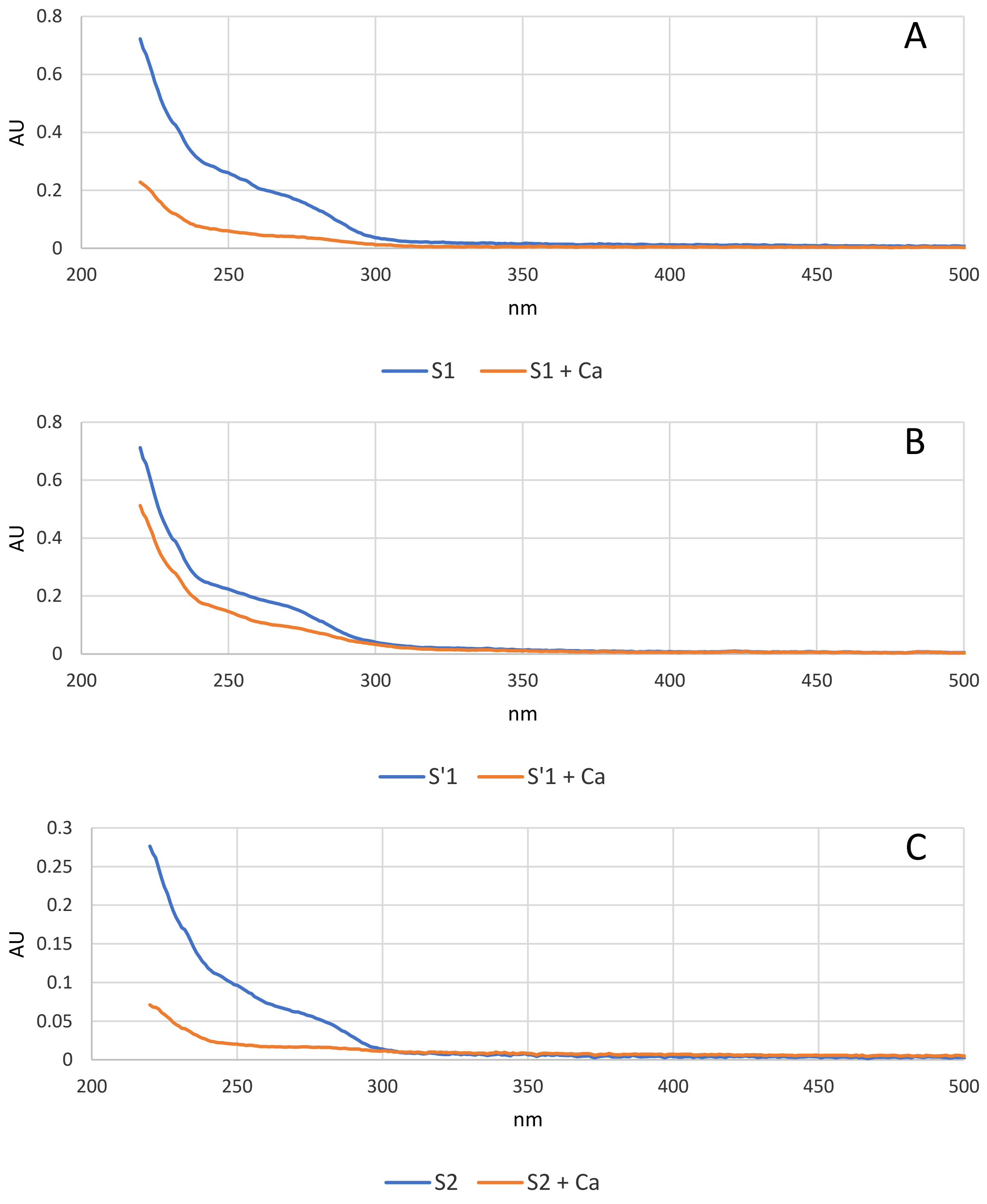

Figure S4: UV-vis spectra at 0.5% w/v of S1(A) S'1(B) S2(C) with and without calcium at 0.1M.

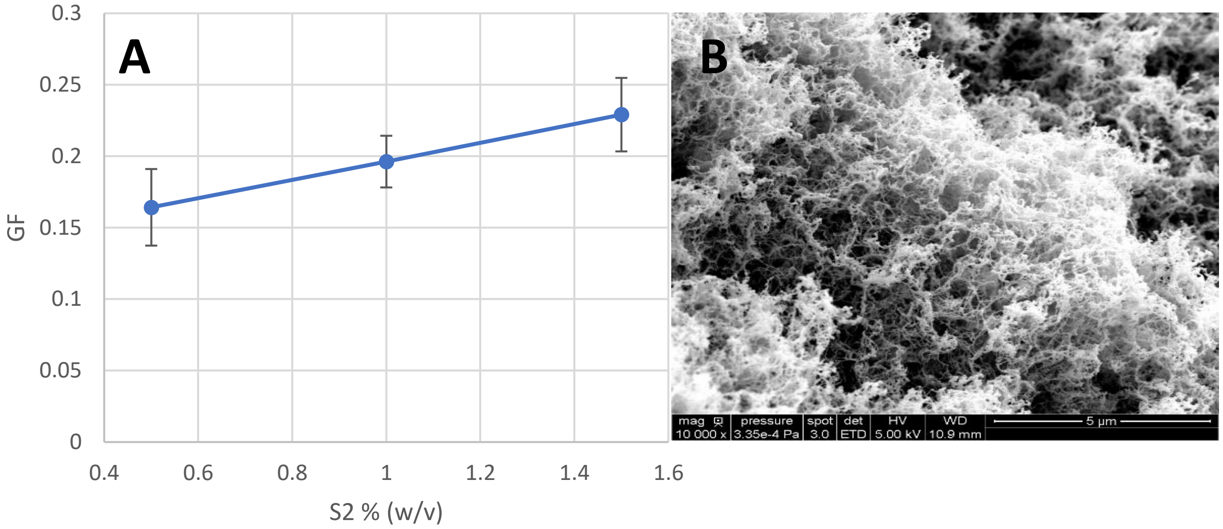

Figure S5: The effect of the concentration % (w/v) of the solutions of S2 (precipitated EPS) on the GF (A) and SEM observation of a 1.5% (w/v) with 0.1 M calcium S2 hydrogel (B).

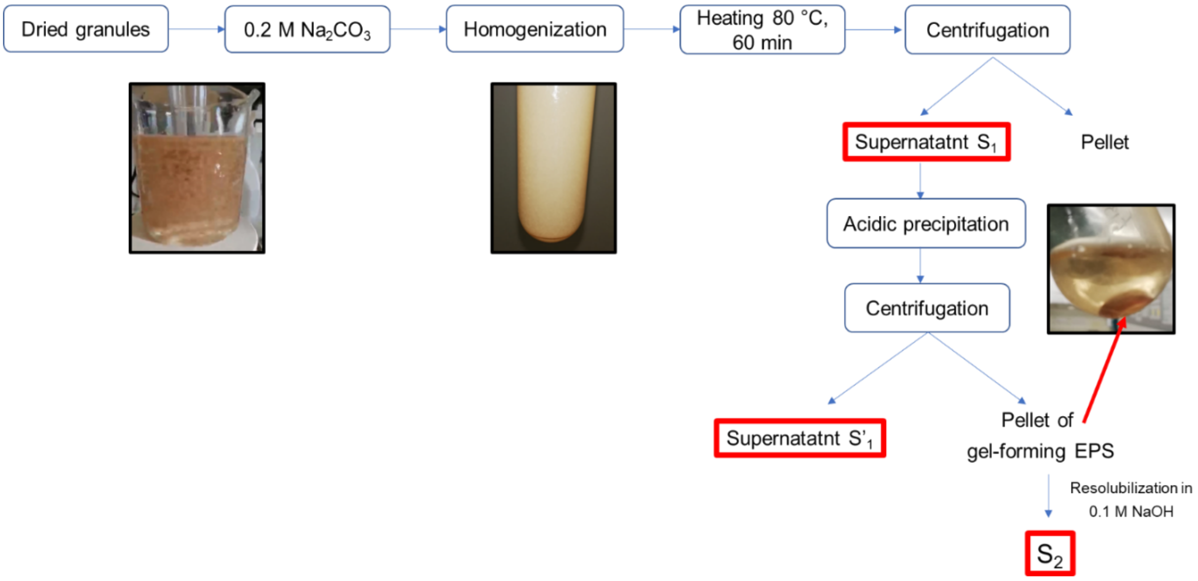

Figure S6: Summary of the extraction protocol of gel-forming EPS.
